# An AI-Supported Methodology for Analyzing Deflections and Misalignments in Human Interactions

**DOI:** 10.1007/s12124-025-09948-x

**Published:** 2025-10-31

**Authors:** Yair Neuman

**Affiliations:** The Functor Lab, Ben- Gurion University of the Negev, Beer-Sheva, 84105 Israel

**Keywords:** Conversation, AI, LLM, SADMA, Misalignment, Speech acts, Noël coward

## Abstract

This paper introduces Speech Act Deflection and Misalignment Analysis (SADMA), an AI-assisted methodology for identifying conversational misalignments that reveal underlying interpersonal dynamics. Grounded in a “meaning-as-a-response” framework—combining Conant’s information theory and Bakhtin’s dialogism—SADMA analyzes utterance-response pairs to detect deflection points where expected conversational trajectories break down. By leveraging a Large Language Model to identify speech acts and goals, SADMA offers objective insights into subjective meaning-making. Applied to Noël Coward’s *Private Lives*, the method highlights patterns of relational conflict and miscommunication. SADMA provides a systematic tool for analyzing conversational breakdowns in psychology, social science, and literary studies.

## Introduction

Noël Coward’s “Private Lives” is a highly successful romantic comedy from the 30s. The comedy describes a divorced couple named Elyot and Amanda. My discussion of the comedy refers to the written play (Coward, 2013) and the BBC TV version from 1976 with Penelope Keith as Amanda and Alec McCowen as Elyot.

While celebrating his honeymoon with his new wife, Sibyl, Elyot surprisingly encounters his ex-wife, Amanda, who arrived at the same hotel in France to celebrate her honeymoon with her new husband, Victor. Although divorced for five years, their meeting revives their love into a stormy and chaotic plot. With its tantalizing ups and downs, this comedy has been intensively studied, with some interpretations wittingly described by Coward as intelligent but “absolute rubbish” (Richards, 1970). Producing an intelligent albeit absolute rubbish is a danger facing the interpretation of any play. This danger is comprehensible when realizing that the shift from the aesthetic-intuitive experience of the play to its analytic understanding is always a challenge, accompanied by the risk of generating interpretations that might be considered “absolute rubbish”. At the same time, while watching the play, one may intuitively grasp patterns of interaction. If patterns of interactions do exist, then it means that some interpretations of the play are more reasonable than others that involve *overinterpretation* (Eco, 1992). The ability to ground this intuition in patterns is a challenge, but a challenge no different from any attempt to scientifically understand the meaning of human interactions. For instance, in the play’s opening scene, Sybil is standing on the balcony, amazed by the view. She says (Coward, 2013, p. 10):

“It’s heavenly, look at the lights of the yacht reflected in the water. Oh dear, I’m so happy.”

Smiling, Elyot responds to this utterance, saying:

Are you?

These two lines of dialogue from the opening scene illustrate both the promise and difficulty of understanding the meaning of an interaction. Elyot’s new wife seems to express her excitement. She has just been married and arrived in France to celebrate her honeymoon. When watching the beautiful view from the balcony, the context of a happy new marriage, a honeymoon in France, and a beautiful view merge into excitement. She shares her excitement with her new husband, who responds with the question: “Are you?” What is the meaning of Elyot’s response? Is it a skeptical comment? A humorous teasing? What does it teach us about how he understood his wife’s utterance? Does it represent a more general pattern of interaction? Moreover, to what extent does this pair of utterances represent a (mis)alignment between the interlocutors?

An alignment exists when there is a correspondence between the conversational moves of the speakers. If Sybil shares her excitement, she probably expects Elyot to *acknowledge* her excitement rather than question her happiness cynically. In a case of misalignment, we can identify a deflection point that may be highly informative for understanding the interlocutors, the interaction patterns, and the couple’s dynamics. This understanding may be highly informative for understanding fractures and crises in interpersonal relationships. Specifically, it may help us better understand the play’s turmoil dynamics, romantic moments, and interpersonal crises.

In this paper, I propose one possible methodology for addressing the challenge of understanding misalignments for understanding interaction patterns and dynamics. This AI-supported methodology is grounded in meaning-as-a-response, which will be discussed in the next section. It is also grounded in innovative ventures using modern AI technology, specifically large language models, for psychology, the social sciences, and the humanities (e.g., Neuman et al., 2023; Neuman, 2024; Neuman, 2025). The proposed methodology, *Speech Act Deflection and Misalignment Analysis (SADMA)*, is presented and illustrated with respect to three examples. It is then applied to Private Lives to illustrate its power in supporting our understanding of the play and interaction patterns in general. Hopefully, interpreting the play’s opening scene through SADMA will show that it is possible to generate intelligent interpretations of the play without the risk of generating “absolute rubbish.”

## Meaning as a Response

In 1979, Roger Conant, a professor of Information Engineering and a pioneer in cybernetic and information theory, published an insightful paper (Conant, 1979) that was hardly noticed. To date, the paper has only eleven citations. This ignorance can be emphasized by comparing the paper to Conant’s most cited paper, which has gained 1802 citations. The title of Conant’s paper is “A vector theory of information.” The title might be misunderstood as representing a purely technical academic paper. However, it was a revolutionary paper that preceded our understanding of *meaning* as expressed, for instance, by the current Large Language Models. Conant’s central thesis surprisingly resonates with the idea of meaning as a response, a pragmatic theory of meaning associated with the work of Bakhtin as interpreted by Holquist (1983). We should carefully understand Conant’s argument to understand this strange alliance between a Russian intellectual and an engineering professor.

The context of Conant’s argument is information theory and its enormous success. Emerging in a limited context of engineering, Shannon’s information theory gained enormous influence beyond its original and limited scope. Conant’s paper first points to the limitations of Shannon’s notion of information in calculating “the information carried by real messages transferred in the real world of human conversation.” Conant convincingly illustrates this point. Suppose he writes, someone is shouting “Fire!”. According to Shannon, the information of this message is reducible to the *probability* of its appearance in human interaction: -log_2_p. However, the meaning of “Fire!” is its “effect on those who hear it.” It is the escape *response* that expresses the message’s meaning and not its probabilistic aspect. The important thing about a *message*, whether intentional or not, is its *meaning*, *which is represented by a corresponding response*. As Conant suggested, information exists in the *relation* between a message and a receiver, and a message carries information if it modifies the receiver’s representation, as evident from his response. The person shouting “Fire!” probably meant to warn other people of a danger. Escaping from the theatre, the audience’s response expresses the utterance’s meaning. Conant further shows how knowledge can be represented through a vectorial representation and how to model knowledge modification through transformations in a vector space. The modern expression of this approach appears in LLMs, where all knowledge is represented as dynamic and contextual representations of knowledge.

Interestingly, Conant’s theoretical approach is aligned with the one presented by Holquist in Dialogism (Holquist, 1983). One of the central ideas Holquist brings out from Bakhtin’s work is that meaning is fundamentally dialogic. Every act of speaking or writing is situated in a *dialogue*, even if the other speaker is imagined. The notion of dialogue is not restricted, of course, to human beings. From the most general perspective, a dialogue is any interaction where messages are exchanged, interpreted, and responded to. In human interaction, when one speaks, they respond to an utterance (i.e., the most basic communication unit). Therefore, the meaning of any utterance is determined in part by how it is answered. In this sense, meaning does not reside solely in intention or the words themselves, but in how others respond to those words. Therefore, in Holquist’s interpretation, to say “meaning is a response” is to highlight that utterances become meaningful *only* in their social context, through the interplay of dialogue, and that the dialogic nature of language makes meaning inherently dynamic and unstable, always in the process of becoming through interaction.

Both Conant and Holquist focus on the process of meaning-making. They focus on the interaction between individuals, how utterances change representations, and how they are responded to. However, despite the theoretical appeal of this approach, there is a lacuna concerning how meaning-making can be “objectively” studied through the mediation of AI. By “objectively”, I do not mean that meaning exists independently of its interaction, an idea that contradicts the basic tenets of the approach used in this paper, but that the interactional and subjective process of meaning-making can be scientifically studied regardless of our own personal bias, inclination, or theories.

## Who Is terrible? The Coffee or the wife?

A famous anecdote, attributed to Paul Watzlawick, is the question: When can couple therapy be terminated? The response is when the husband says to the wife, ‘This coffee is terrible,’ and they BOTH know that he is talking about the coffee. We can use this situation to explain how interactional meaning can be objectively analyzed in context. Assume a husband and a wife having their breakfast during the 50s. The husband tastes the coffee and says, “The coffee is terrible.” In response, the wife shouts, “You are such an ungrateful swine!” In itself, and given the limited context of the example, we do not understand the meaning of the husband’s original utterance. His mental state is beyond our reach. His intention may have been to point out that the coffee tastes terrible because it is bitter. Alternatively, he may have intended to argue that his wife is terrible because she does not know how to prepare a decent cup of coffee. It is not trivial to decide which of these two possible intentions the utterance expresses. However, through the wife’s response, we can understand that *she* interpreted the utterance as negatively loaded and probably as a criticism. The idea of meaning as a response allows us to go beyond mental structures and intentions and to study objectively how meaning is formed “in-between” individuals. Nevertheless, there is a shortage of methodologies that can support this scientific understanding. In this paper, I propose an AI-supported methodology that may help researchers to address this challenge. The methodology is illustrated through the analysis of three worked examples.

## Speech Act Deflection and Misalignment Analysis (SADMA)

### Example 1. The Coffee Is Terrible

The methodology focuses on utterance-response pairs. It can include the relevant context of the conversation using the approach described in Neuman (2024). For simplicity, I start with a decontextualized example drawing on Watzlawick’s insightful suggestions. The example I use is an imagined two-line dialogue between a husband and a wife. I specifically designed this two-line dialogue to express an extreme form of misalignment. This extremity has a didactic aim. Nevertheless, it corresponds with extreme cases of interpersonal interactions, such as those that appear in Private Lives. The dialogue is:

Husband: “The coffee is terrible.”

Wife: “Fuck you!”

To understand the meaning of the husband’s utterance, I analyze the wife’s utterance. My focus is on speech acts, a powerful concept that is highly constructive in analyzing human interactions through AI (e.g., Neuman, 2024; Neuman, 2025). First, we automatically identify the utterance’s speech act, conversational goal, and expected response. For example, when the husband says “The coffee is terrible”, his utterance may express three main speech acts/conversational goals. The first is a complaint/criticism. By pointing to the sour taste of the coffee, the husband may complain about its taste or about his wife, who served him such coffee. A second possible speech act is an “Indirect request.” By describing the terrible taste of the coffee, the husband may indirectly ask his wife to prepare him another cup of coffee that tastes better. Finally, the utterance may express the speech act of *warning*, advising his wife to avoid drinking the coffee that tastes horrible. Given a lack of context, the most reasonable interpretation of the utterance is that it expresses a complaint/criticism. However, we can include context in our analysis by simply using the following prompt, where the term < CONTEXT > directs the model to any relevant contextual information.

### Prompt. Speech Acts Analysis

A speech act is a fundamental unit of communication where an utterance acts.

Analyze the following utterance: Husband: The coffee is terrible.

To analyze the utterance, use < CONTEXT>.

Instructions:

Identify the three most likely speech acts expressed by the utterance.

Ensure you present three different speech acts.

Present your answer as the titles of the three speech acts only (e.g., complaint/criticism).

Among the three, identify the speech act most likely expressed by the utterance.

Given the identified primary communicative function, we then identify the most likely response to the utterance. For instance, if the husband complains, a possible response is problem-solving, such as in the utterance: “Let me prepare a better coffee”. We then move to the actual response analysis by analyzing the wife’s response. By responding “Fuck you!” the wife uses a *Personal attack*. The focus of this response is interesting because it completely ignores the coffee and targets the husband personally. In terms of a response strategy, it is an aggressive deflection that transforms a complaint/criticism into a personal confrontation. From the wife’s response, we understand that the meaning she attributes to her husband’s utterance is *a criticism/complaint that is probably directed* at her rather than the coffee. At this point, we may perform alignment analysis to realize that the wife’s response is completely misaligned with her husband’s utterance. When performing a Deflection Pattern Identification, we notice that the response shifts from coffee quality (object) to personal attack (subject), and instead of engaging with mild dissatisfaction, it escalates to intense anger. It avoids any engagement with the complaint by making the husband the problem. This shift expresses a well-known move in communication (Watzlawick et al., 2011), and it is clearly indicative that the couple is having a problem. Instead of BOTH knowing that the husband is talking about the coffee, the wife responds as if it is clear that he is talking about her. Concerning the husband, it is not clear whether he is talking about the coffee or about his wife. The most important aspect concerns the relationship part. The wife is using a defensive and explosive communication style. Regarding power dynamics (Neuman & Cohen, 2024), the wife asserts dominance through aggression, shutting down the complaint. We can hypothesize that, underlying her response, there is a significant tension and a pre-existing marital conflict. The focus here is on one aspect of the interaction: the misalignment between the interlocutors and their interpersonal meaning. To formalize this procedure, we can use the following general prompt:

### Speech Act Deflection and Misalignment Analysis Prompt Instructions

Use this prompt to systematically identify when a response deflects from or misaligns the expected speech act sequence.

#### Analysis Framework

**The given data is**:

Context: [Insert context here]

Utterance: [Insert utterance here]

Response: [Insert response here]

#### Step 1: Expected Response Mapping

For the initial utterance, identify:

- **Primary speech act**: What is the main communicative function?
- **Expected response type**: What speech act would typically follow? (e.g., agreement, acknowledgment, reciprocation, compliance)
- **Conversational goal**: What is the speaker trying to achieve interpersonally?

#### Step 2: Actual Response Analysis

For the response utterance, determine:

- **Actual speech act performed**: What did the responder do?
- **Identify the speech act the actual response typically responds to**: To which speech act does *the s*peech act you identified in the previous step usually respond?
- **Response focus**: What element of the initial utterance (if any) does the response address?
- **Response strategy**: Does it engage with, avoid, redirect, or challenge the initial speech act?

#### Step 3: Alignment Assessment

Compare expected vs. actual:

- **Direct alignment**: Does the response fulfill the expected speech act? (Yes/No)
- **Partial alignment**: Does it address some aspect while deflecting from others?
- **Complete misalignment**: Does it ignore the expected response entirely?

#### Step 4: Deflection Pattern Identification

If misalignment occurs, categorize the deflection type:

- **Topic deflection**: Shifts focus to different subject matter.
- **Emotional deflection**: Avoids engaging with expressed emotions.
- **Authenticity deflection**: Questions sincerity rather than engaging with content.
- **Responsibility deflection**: Redirects accountability or focus.
- **Temporal deflection**: Shifts from present to past/future concerns.

#### Step 5: Relationship and Context Implications

Consider what the deflection reveals:

- **Power dynamics**: Who controls the conversational direction?
- **Intimacy level**: Does deflection suggest comfort with probing OR distance/avoidance?
- **Conflict indicators**: Does misalignment signal underlying tension?
- **Communication style**: Is this deflection habitual or situational?

### Output Template

Produce a short title/response to each point, for steps 1 to 5.

Here is the application of this general prompt to the coffee example. I used ChatGPT and Claude for all prompts used in this paper. The output produced for the coffee example is:

## Application of Deflection Analysis Prompt

### Analysis Framework

#### Step 1: Expected Response Mapping

Initial Utterance: “The coffee is terrible.”

- **Primary speech act**: Complaint/Criticism (about the coffee quality).
- **Expected response type**:
  - Acknowledgment (“Yes, it is bad”).
  - Problem-solving (“I will make a fresh pot”).
  - Apologetic explanation (“Sorry, I must have made it too strong”).
  - Disagreement (“I think it tastes fine”).
- **Conversational goal**: Expressing dissatisfaction and implicitly seeking either validation of the complaint or a remedy.

#### Step 2: Actual Response Analysis

Actual Response: “Fuck you!”

- **Actual speech act performed**: Hostile rejection/Personal attack.
- **The speech act to which the actual response responds**: Personal criticism/attack on her competence or worth as a partner (interpreting the coffee complaint as an implicit judgment of her domestic abilities or general adequacy).
- **Response focus**: The response completely ignores the coffee and targets the husband personally.
- **Response strategy**: Aggressive deflection - transforms a product complaint into a personal confrontation.

#### Step 3: Alignment Assessment

Compare expected vs. actual:

- **Direct alignment**: No - the response does not address the coffee quality at all.
- **Partial alignment**: No - no aspect of the complaint is acknowledged or addressed.
- **Complete misalignment**: Yes - entirely ignores the stated concern and escalates to personal hostility.

#### Step 4: Deflection Pattern Identification

**Deflection Type**: **Topic + Emotional deflection**.

- **Topic deflection**: Shifts from coffee quality (object) to personal attack (subject).
- **Emotional deflection**: Instead of engaging with mild dissatisfaction, it escalates to intense anger.
- **Responsibility deflection**: Avoids any engagement with the complaint by making the husband the problem.

#### Step 5: Relationship and Context Implications

- **Power dynamics**: Wife asserts dominance through aggression, shutting down the complaint.
- **Intimacy level**: Suggests significant underlying tension - this level of hostility over a minor complaint indicates deeper issues.
- **Conflict indicators**: Strong signal of pre-existing marital conflict; the coffee complaint likely triggered accumulated resentment.
- **Communication style**: Defensive, explosive pattern that prevents problem-solving.

#### Application Results

**Expected Response:** Acknowledgment of coffee quality or problem-solving response.

**Actual Response:** Personal attack/hostile rejection.

**Alignment Status:** Complete misalignment.

**Deflection Type:** Topic + Emotional deflection with aggressive escalation.

**Interpersonal Impact:** This deflection pattern suggests a relationship under severe stress where even minor complaints trigger disproportionate hostility. The wife’s response indicates she may interpret routine complaints as personal attacks, suggesting either accumulated resentment or a deeper communication breakdown. This deflection prevents practical problem-solving and transforms mundane issues into relationship conflicts.

The extreme nature of this misalignment (from coffee critique to profanity) indicates the conversation was never really about coffee - the wife is responding to something else entirely, possibly feeling criticized, unappreciated, or overwhelmed in the relationship.

Using SADMA, we learned that AI guided by theory can perform a valid and automatic analysis of misalignments and identify deflection points in a conversation. The output produced by SADMA is automatically generated and can support a valid interpretation of conversations by specifically trying to understand interpersonal crises and how they evolve.

### Second Example: Two Embarrassed Men and an Angry Woman

My second example concerns a very short humorist sketch by Hanoch Levin. Levin was Israel’s most important and influential playwright, known for his sharp, often provocative combination of dark humor, existential despair, and social critique. Here, I use *The Gigolo from the Congo and Other Characters* (Levin, 1994), which is a collection that includes short, absurdist sketches or monologues, often darkly comic and grotesque. These pieces reflect Levin’s signature blend of existential despair, bodily humor, and brutal emotional honesty.

The short sketch I use is titled: “Two embarrassed men and an angry woman.” The context is a husband, wife, and a guest who are seated at the table eating. The guest is the first to speak, asking: “Excuse me, could you please pass the salt?” This utterance echoes Searle’s classical example (Searle, 1969), illustrating the illocutionary force of an utterance (i.e., what kind of act is being performed in saying something). The second utterance is by the husband, asking his wife to pass the salt:

**Husband: **Genia, could you please pass the salt?

From this utterance, we infer that the wife did not respond to the request by the guest. However, the wife does not respond to her husband’s utterance, and he repeats the request:

**Husband:** Excuse me, Genia, could you please pass the salt?

This second utterance involves a repetition of the first, plus a polite approach (i.e., “excuse me”). At this point, the wife responds, and all hell breaks loose. Her reaction is explosive. She erupts with a long, furious monologue, accusing her husband of being weak, emasculated, soft, even impotent, attacking his masculinity in a grotesquely vivid and darkly comedic fashion:

**Wife:** Why are you barking?! What’s with this “Excuse me, Genia” nonsense?! Why are you yelling at me like I’m a dog?! “Salt”! You’re shouting! Opening that big mouth of yours! We’ve seen your type before! “Pass the salt”! A man! Very small! Very small and very soft and not firm! So you open your mouth! “Excuse me, Genia”! And “Would you mind?“! And “Pass the salt”! “The salt”?! – Soft and small and not firm and doughy – that’s what it is, not “the salt”!

And her monologue continues ….

The wife’s response has nothing to do with the request. She accuses her husband of “barking”, yelling at her like she is a dog, and then blames her husband of impotence. Her response is totally misaligned with the husband’s utterance. A rather trivial request is responded to with a humiliating personal attack and mockery. This sketch expresses well-known themes in the plays and sketches of Levin, such as the way in which a mundane domestic exchange can turn into a grotesque, explosive psychological confrontation. The sketch also includes humiliation, sexual frustration, power imbalance, and the raw cruelty often found in intimate relationships. Though the sketch is minimalist on stage, its emotional and rhetorical force is maximal as the wife’s tirade shatters the facade of polite bourgeois decorum.

Let me apply SADMA to analyze the meaning of the wife’s response. In the < CONTEXT>, I include all background information until the husband’s second utterance:

Context:

A husband, wife, and guest sit at a table and eat.

Guest: Excuse me, could you please pass the salt?

Husband: Genia, could you please pass the salt?

[The wife does not respond]

Using ChatGPT, I produced the following analysis of the husband’s utterance and his wife’s response:

### Initial Utterance

**Primary Speech Act: **Request.

**Expected Response Type: **Compliance or refusal (typically compliance in polite company); minimal acknowledgment would also be acceptable (e.g., passing the salt, saying “Here you go”).

**Conversational Goal:** Maintain social decorum and hospitality by fulfilling a simple request from a guest; facilitate smooth interaction and polite behavior.

The first analysis identifies the husband’s utterance as a *request* inviting compliance. Then, SADMA analyzes the actual response:

### Actual Response

**Actual Speech Act Performed:** Verbal confrontation and personal attack.

**The Speech Act To Which the Actual Response Responds:** Domineering command/emasculating public humiliation (interpreting the polite request as an aggressive, demeaning order that exposes his weakness and inadequacy as a man).

**Response Focus:** Superficially on the husband’s tone/language (“Excuse me, Genia”), but rapidly escalates into broader grievances, including emotional complaints, emasculation, and sexual criticism.

**Response Strategy:** Challenges and redirects; initially questions the husband’s speech act tone, then redirects to broader emotional and personal dissatisfaction.

The analysis points to the meaning Genia (i.e., the wife) attributes to her husband’s innocent request. Her response is as if her husband is trying to dominate and humiliate her by asking for salt. SADMA validly assesses the alignment between the utterances:

**Alignment Assessment**.

**Direct alignment: No**.

**Partial Alignment**: **No**

**Complete misalignment: Yes**.

The response does not acknowledge, comply with, or engage the literal request; it instead initiates a separate, emotionally charged monologue that attacks the husband’s character and past behavior.

It suggests a complete misalignment, and then the deflection analysis:

#### Deflection Pattern Identification

**Deflection Types Present**:

1. Emotional deflection:

- Avoids responding to the actual request by expressing resentment and reactivity to perceived condescension and past emotional injuries.

2. Responsibility deflection:

- Positions herself as the wronged party, refusing to engage with the cooperative frame of the initial request by reframing it as an attack.

3. Topic deflection:

- Shifts the conversation from the immediate practical request (“pass the salt”) to a critique of the husband’s character, past sexual behavior, and tone.

4. Authenticity deflection:

- Calls into question the sincerity of the husband’s request by mocking his choice of words (“‘Excuse me, Genia’ nonsense”).

SADMA identifies deflection concerning the four types of deflection. The relationship and context implication deriving from this analysis is:

#### Relationship and Context Implications

**Power dynamics**:

The wife seizes control of the conversational floor through a lengthy and emotionally dominant monologue, asserting power over the interaction by refusing to participate in the expected cooperative exchange.

**Intimacy level**:

Indicates **high familiarity but strained intimacy**—the wife feels entitled to voice deep, unresolved grievances in front of a third party, suggesting a breakdown of private boundaries and emotional safety.

Conflict indicators:

**Strong indicators of underlying and possibly chronic conflict**—the disproportionate response signals unresolved emotional tension, possibly resentment or long-term dissatisfaction in the marriage.

**Communication style**:

**Habitual or situational?**

The emotional intensity and speed of escalation suggest this may be **habitual**, possibly part of a broader dysfunctional communication pattern in the relationship.

SADMA summarizes the analysis as follows:

**Alignment Status:** Complete misalignment.

**Deflection Type(s):** Emotional, Responsibility, Topic, and Authenticity deflection.

**Interpersonal Impact**:

The interaction reveals deep-seated marital tension, power struggles, and a breakdown in cooperative communication. It suggests that even neutral or polite speech acts can be reframed as hostile due to accumulated grievances, pointing to a conflict-laden dynamic masked by social routines.

In sum, SADMA produced a valid analysis of the interaction between the husband and the wife. It identified a misalignment with emotional deflection types and a deep-seated marital tension between the husband and the wife. We can see that the analysis produces valid outputs with respect to the two extreme examples. However, the third example challenges the methodology with a more delicate situation,

#### Third Example: Private Lives

At this point, we may return to Private Lives and test the prompt on the two lines of dialogue discussed in the introduction. SADMA produces the following analysis of Sybil’s utterance, identifying her emotional self-disclosure and her expectation for affectionate acknowledgment:

**Step 1: Expected Response Mapping**

**Primary Speech Act:** Emotional self-disclosure/expressive.

**Expected Response Type:** Affectionate acknowledgment, emotional reciprocation, or supportive alignment (e.g., “Me too,” “It’s beautiful,” “I’m happy you’re happy”).

**Conversational Goal:** To share joy, affirm emotional connection, possibly elicit emotional or aesthetic resonance from partner.

The analysis of the actual response by Ely exposes the meaning that he attributes to Sybil’s utterance and the misalignment between the husband and his wife:

**Step 2: Actual Response Analysis**

**Actual Response**:

**Husband:** “Are you?”

**Actual Speech Act Performed:** Indirect challenge or questioning of sincerity.

**The Speech Act To Which the Actual Response Responds:** The wife’s emotional self-disclosure (“I’m so happy”).

**Response Focus:** Directly addresses the emotional claim, but questions its authenticity rather than affirming or aligning with it.

**Response Strategy:** Challenges or redirects emotional content; minimal engagement.

Instead of affectionate acknowledgment, Ely questions his wife. In terms of alignment:

**Step 3: Alignment Assessment**.

**Direct alignment: No**.

**Partial alignment: No** – while the response refers to the emotion, it does not affirm or share it.

**Complete misalignment: Yes** – it counters the expected supportive or affiliative response with skepticism.

We can see that his response counters the support and acknowledgment expected by his wife. This misalignment generates a point of deflection:

**Step 4: Deflection Pattern Identification**

**Deflection Type: Authenticity deflection**.

**Description:** The husband avoids reciprocating the emotional content and questions whether her statement is genuine. This casts doubt on her emotional state rather than engaging with it as sincere.

Ely questions the authenticity of Sybil’s emotion; the meaning of his response is indicative of mistrust. Sybil expresses positive excitement, which Ely interprets as inauthentic. SADMA analyzes the relationships and context implications as follows:

**Step 5: Relationship and Context Implications**

**Power Dynamics:** The husband seizes control of the emotional tone, disrupting the wife’s expressive moment. This suggests an imbalance where emotional vulnerability is not equally supported.

**Intimacy Level:** Suggests **emotional distance**; the husband is either uncomfortable with intimacy or unwilling to participate in emotional attunement.

**Conflict** **indicators: Yes** – subtle tension is indicated; the husband’s response may reflect skepticism, cynicism, or prior unresolved emotional issues.

**Communication Style:** The husband may have a **habitual or situational avoidance** of emotional engagement, possibly signaling more profound relational disconnection or discomfort with affective displays.

What can we learn from the automatic analysis generated by SADMA so far? At this point, and with such a short exchange, we cannot reach conclusions but generate hypotheses. The first concerns the characters. Sybil seems to be an emotionally expressive woman who seeks acknowledgement. Ely seems to be emotionally distant, cynical, and distrustful. Their relationship pattern is misaligned with an emotional deflection point: the wife seeks acknowledgment, and the husband seeks distance. This AI-supported reading of the exchange can be extended to the first exchange in the play, as elaborated in the next section.

### Analyzing the First Exchange from Private Lives

Given the abovementioned example, we can run the analysis on the first exchange from the movie:

[A grand beachfront hotel at Le Touquet, France]

1. Wife: “Ely…! Ely, dear, do come out! It’s so lovely!”
2. Husband: “Just a minute! Not so bad!”
3. Wife: “It’s heavenly! – Look at the lights of that yacht, reflected in the water! – Hmm! Oh, dear, I’m so happy!”
4. Husband: “Are you?”
5. Wife: “Aren’t you?”
6. Husband: “Oh, of course I am! Tremendously happy!”
7. Wife: “Just to think,… …here we are, you and I, married!”
8. Husband: “Yes, things have come to a pretty pass!”
9. Wife: “Don’t laugh at me! You mustn’t be blasé about honeymoons, just because this is your second!”

Running SADMA, we can try to understand this short interaction. All outputs of SADMA appear in the appendix, and my analysis and interpretation draw on these outputs. We start by analyzing the first two utterances. Sybil invites Ely to enjoy the scenery, expecting compliance/agreement. She aims to share her joy. Ely delays compliance (“Just a minute!”) and provides a judgment of the scene (“Not so bad”). He acknowledges the content (the view) but reframes the scenery as moderately appealing—nothing to be excited about. The alignment between the utterances is partial. It addresses the setting (“Not so bad”) and acknowledges the utterance (“Just a minute”) but does not reciprocate enthusiasm or act on the invitation. The deflection is mostly emotional as Ely avoids matching the emotional enthusiasm expressed by his wife. There is a misalignment between the “lovely” and “Not so bad”. Ely controls the situation by moderating his wife’s excitement and emotionally distancing himself from the event.

Given her husband’s moderate response, Sybil repeats and amplifies her excitement (“It’s heavenly! “), emphasizing her high emotional state (“I’m so happy!”), again, seeking confirmation from Ely. However, he questions her happiness in a way that is used to address factual or exaggerated claims by asking, “Are you?”. It seems that Ely is trying to turn her off, for the second time. Lovely responds with “Not so bad”, and happiness is questioned. A husband who consistently “turns off” his wife this way could be described as emotionally dismissive or invalidating. It is a behavior that undermines emotional intimacy. By dismissing what matters to Sybil, he creates emotional distance. The behavior also signals a lack of appreciation instead of affirming or acknowledging her perspective; he devalues it. Finally, it can feel competitive or one-upping – implying that her experience or judgment is not good enough.

The meaning Ely attributes to Sybil’s utterance is therefore of an exaggerated claim that should be undermined and downplayed. Therefore, his response undermines Sybil’s emotional statement instead of aligning with it. This response expresses misalignment as it negates the expected support and introduces doubt or emotional detachment. Again, we observe an *emotional deflection* where Ely avoids emotional affirmation and possibly reflects discomfort with emotional intimacy. Regarding relations, we can see that Ely takes control of the emotional tone, possibly asserting a cooler or more distant stance. Moreover, his response may suggest disbelief, sarcasm, or disconnection, hinting at a strain or unresolved tension in their dynamic. His style leans toward coolness or ironic detachment, contrasting with the wife’s effusive openness. We may hypothesize that Ely is a character of low empathy, insecurity, and need for superiority, critical/judgmental thinking style, defensive/dismissive coping strategies, and that he expresses possible narcissistic traits.

To Ely’s “question”, Sybil responds with the same question. Instead of affirming or explaining her feelings, Sybil redirects attention back to Ely. She interprets Ely’s question as expressing doubt and responds via emotional mirroring and deflection. She does not elaborate on her feelings, confirming she is happy, but challenges him to match them. Therefore, her response expresses partial alignment as it engages emotionally and keeps the affective tone but sidesteps the question of her own emotion in favor of assessing his. Therefore, Sybil’s response expresses two types of deflection. Responsibility deflection, where she shifts emotional responsibility onto the husband by turning the question back, and emotional redirection, where she seeks emotional validation or parity instead of providing self-validation. Her response is indicative of vulnerability and insecurity. She is seeking acknowledgment, and when she does not get it, she anxiously turns to Ely, checking his stance. While Ely seems a narcissist seeking power and significance, Sybil may be a woman seeking acceptance from a powerful and significant other (i.e., her husband).

Sybil is seeking emotional connection, even if through indirect means. Ely’s response is analyzed in output 4. He presents an emphatic affirmation with intensification (“Oh, of course I am! Tremendously happy!“), showing complete alignment with Sibyl. However, given the previous analysis, we may question the authenticity of his exaggerated response. He is “tremendously happy”, and the gap between his previous dismissive and cynical approach and his use of the strong adjective “tremendous” calls into question the authenticity of his response. However, Sybil does not question the authenticity of his exaggerated response. The authenticity issue is resolved when Sybil says:

“Just to think,… …here we are, you and I, married!”

Ely responds by saying:

“Yes, things have come to a pretty pass!”

The phrase “things have come to a pretty pass!” is an idiomatic expression meaning that circumstances have reached an unfortunate, troublesome, or undesirable state. It often expresses dismay or irony about how badly a situation has deteriorated. In this context, when Ely says this in response to Sybil’s romantic reflection about their marriage (“Just to think… here we are, you and I, married!“), he says their marriage represents a sorry state of affairs or an unfortunate turn of events. His utterance carries a tone of resignation mixed with complaint, as if he is saying, “Well, this is what we have come to” or “Look what a mess we have gotten ourselves into.” It is particularly cutting because it reframes what his wife sees as a romantic moment (celebrating their union) as something regrettable or problematic. This is the third instance where Ely is dismissing his wife, misaligning with her emotions, and forming an emotional point of deflection. In other words, Sybil is seeking intimacy through affectionate reflection. However, Ely responds as if she describes a problematic situation. His move expresses complete misalignment as he ignores her intimate intent and reframes the positivity of her romantic utterance as negative. He undermines her emotional vulnerability with a dismissive response and retreats from an intimate moment through sarcasm, suggesting underlying marital dissatisfaction. His defensive and deflecting communication style indicates emotional unavailability or relationship strain.

Sybil’s final utterance (“Don’t laugh at me! You mustn’t be blasé about honeymoons, just because this is your second!“) is analyzed in output 6. She uses a defensive rebuke with a personal accusation about Ely’s past experience. Her response indicates that she conceives Ely’s utterance as a mockery, and that she aims to address this perceived mockery and attribute his attitude to his marital history. Her response presents partial misalignment as she responds to the negative tone but misinterprets the target. In terms of deflection, the following types are expressed:

Responsibility deflection: Shifts blame to husband’s past rather than addressing present complaint.

Emotional deflection: Avoids engaging with his expressed dissatisfaction about their situation.

Temporal deflection: References past marriage to explain present attitude.

Regarding the relationship, Sybil expresses vulnerability while attacking her husband’s credibility. She reveals insecurity about being his second wife and shows high tension, underlying jealousy, and defensive positioning. The communicative style, accusatory and defensive, suggests deep-seated insecurities about their relationship.

Summarizing the analysis performed by SADMA, we can generate our understanding of the characters, their relational pattern, and dynamics. Sybil is an expressive and emotionally involved character seeking acknowledgment from her husband. Regarding known personality types (Lingiardi & McWilliams, 2017), we may hypothesize that she is a dependent personality whose most basic need (Huges, 2020) is *acceptance*. Her responses are indicative of insecurity and vulnerability. In contrast, Ely seems to be a narcissist seeking to express his superiority and power, while exposing his own vulnerability and emotional distancing. The relational pattern is mostly asymmetrically misaligned. Even in this short interaction, Ely is completely misaligned with Sybil, presenting mostly emotional deflection points. Among the four responses he generates, two present complete misalignments, one presents partial misalignment, and one is allegedly complete alignment (Tremendously happy!). He mainly presents emotional points of deflection and moderate to low expressions of intimacy. The meaning he attributes to Sybil’s utterances expresses his distorted representations. Sybil’s emotional expressiveness and need for acceptance and acknowledgment are interpreted as factual statements whose authenticity can be questioned or interpreted negatively. In terms of meaning-as-a-response, Ely’s responses to Sibyl’s are highly diagnostic of his character and relational patterns. Sibyl is more moderate in her alignment, which is partial primarily, and her points of deflection mostly involve shifting the responsibility to Ely, a move which is in line with her interpretation as a dependent character. However, unlike Ely, her meaning-making is less distorted and more realistic. When she responds, she usually responds in a way aligned with the communicative aim of Ely. The allegedly happy couple arriving in France to celebrate their marriage exposes their personalities, relational patterns, and problematic dynamics. The seeds of the crisis appear in the opening interaction, which can be structurally and validly analyzed through SADMA.

## Discussion

The short exchange, opening Private Lives, is diagnostic regarding exposing the characters and their relational patterns. Sybil is excited about her honeymoon and quite happy about it. However, Ely cannot align with her feelings and positivity. He questions the authenticity of her emotions, expressing deep mistrust, and sarcastically responds and frames the situation, ignoring basic norms of politeness. This short exchange exposes a deep vulnerability in the new couple’s marriage. While presenting cheerfulness and optimism, Sybil acknowledges that there is a problem and that the problem is with Ely’s previous marriage. Her defensive response acknowledges the mockery of his utterance but shifts the responsibility from Ely to his previous marriage. This context allows us to understand Ely’s encounter with his previous wife, Amanda. However, beyond the context of the particular play, SADMA gives us a structured and automatic methodology for analyzing interactions and better understanding points of deflection. The methodology’s prompt is not final and can be easily adapted to various contexts. Its importance, though, is in providing a structural approach for analysis of various interactions, which can generate reasonable interpretations while avoiding “intelligent rubbish”.

## Data Availability

No datasets were generated or analysed during the current study.

## Appendix 1. SADMA’s Outputs

SADMA OUTPUT 1.

Step 1: Expected Response Mapping

- **Primary speech act**:

**Invitation/Exhortation** – The wife invites Ely to enjoy the scenery outside

- **Expected response type**:

Compliance or Agreement – Typically, one would expect a confirming response like “Coming!” or “Yes, it looks beautiful!”

- Conversational goal:

Shared Enjoyment and Emotional Connection – The speaker attempts to co-create a moment of pleasure and connection through shared experience.

**Step 2: Actual Response Analysis**

- **Actual speech act performed**:

**Delay + Evaluation** – Ely delays compliance (“Just a minute!”) and provides a judgment of the scene (“Not so bad”).

- **Speech act typically responds to**:

An Informative or Descriptive Observation – The evaluation (“Not so bad”) is more suited to a commentary, not an invitation. The delay, meanwhile, responds to a request for action.

**Response focus**:

**Partial – Acknowledges the content (the view)**,** but avoids the relational request to join.**

- **Response strategy**:

Avoids and Redirects – It indirectly acknowledges the appeal but postpones action and reframes the scenery as moderately appealing.

**Step 3: Alignment Assessment**

- **Direct alignment**:

**No** – The response does not fulfill the expected compliance or emotional reciprocation.

- **Partial alignment**:

**Yes** – It addresses the setting (“Not so bad”) and acknowledges the utterance (“Just a minute”) but does not reciprocate enthusiasm or act on the invitation.

- **Complete misalignment**:

**No** – It is not entirely unrelated; it engages minimally.

**Step 4: Deflection Pattern Identification**

- **Deflection type**:

**Emotional deflection** – Avoids matching the emotional enthusiasm expressed by the wife.

**Temporal deflection** – “Just a minute!” delays action and shifts engagement to the future.

**Step 5: Relationship and Context Implications**

- **Power dynamics**:

Mild Assertion of Control – Ely asserts control over timing and engagement, subtly resisting the wife’s framing of the moment.

- **Intimacy level**:

Moderate Intimacy with a Hint of Distancing – Comfortable enough to not respond warmly; suggests habitual or momentary emotional distance.

**Conflict** **indicators**: 

**Low-level tension** – The lukewarm tone could signal mild reluctance or disinterest, possibly indicating underlying discord or emotional investment differences.

- **Communication style**:

Habitual Emotional Reserve – Ely’s style may involve understated affect and minimal verbal reciprocity.

**Conclusion Title**:

**Subdued Deflection: Emotional Minimization and Temporal Delay in Response to Enthusiastic Invitation**.

SADMA OUTPUT 2.

**Step 1: Expected Response Mapping**.

- **Primary speech act**:

Emotive declaration/Expressive – The wife expresses joy, awe, and emotional fulfillment.

- **Expected response type**:

Affirmation, Shared emotion, or Supportive Acknowledgment – E.g., “It really is!” or “I’m glad!” or even mirroring her delight.

- **Conversational goal**:

**Emotional bonding** – The speaker wants to share a peak emotional moment and likely seeks validation, intimacy, or empathy from her partner.

**Step 2: Actual Response Analysis**.

- **Actual speech act performed**:

Questioning/Appraisal Challenge – The husband responds with a question that casts a subtle doubt or prompts re-evaluation of her emotional claim.

- **Speech act typically responds to**:

Factual or Exaggerated Claim – Such a questioning tone more commonly follows an assertion requiring clarification or skepticism.

- **Response focus**:

**Targets the emotional declaration (“I’m so happy”) rather than the environmental description (“Look at the lights”).**

- **Response strategy**:

Challenges/emotionally Distances – The response resists or tests the wife’s emotional statement instead of aligning with it.

**Step 3: Alignment Assessment**.

- **Direct alignment**:

**No** – The response does not validate or reciprocate her emotional expression.

- **Partial alignment**:

**No** – While it references her emotion, it questions it rather than supporting or engaging with it positively.

- **Complete misalignment**:

**Yes** – The response negates the expected support and introduces doubt or emotional detachment.

**Step 4: Deflection Pattern Identification**.

- **Deflection type**:

**Emotional deflection** – Avoids emotional affirmation and possibly reflects discomfort with emotional intimacy.

**Authenticity deflection** – The questioning implies a possible doubt about the sincerity or depth of her stated happiness.

**Step 5: Relationship and Context Implications**.

- **Power dynamics**:

Assertive or Emotionally Withholding – The husband takes control of the emotional tone, possibly asserting a cooler or more distant stance.

- **Intimacy level**:

Diminished Intimacy or Emotional Mismatch – His reluctance to join in emotional expression suggests distance or discomfort with shared vulnerability.

**Conflict indicators**: 

Potential Emotional Dissonance – His response may suggest disbelief, sarcasm, or disconnection, hinting at a strain or unresolved tension in their dynamic.

- **Communication style**:

Emotionally Reserved or Probing – His style leans toward coolness or ironic detachment, contrasting with the wife’s effusive openness.

**Conclusion Title**:

**Emotional Disalignment: Authenticity Challenge in Response to Joyful Declaration**.

SADMA OUTPUT 3.

**Step 1: Expected Response Mapping**.

- **Primary speech act**:

Interrogative turn/Emotional Reciprocity Bid – The husband’s “Are you?” is a skeptical or curious query, prompting the wife to reflect or defend her emotional state.

- **Expected response type**:

Affirmation or Elaboration – A typical reply might be “Yes, I really am” or an explanation of her feelings.

- **Conversational goal**:

Emotional Clarity or Connection Reestablishment – The question invites self-affirmation or a mutual understanding of her feelings.

**Step 2: Actual Response Analysis**.

- **Actual speech act performed**:
- **Conversational redirection/Counter-question** – Instead of affirming or explaining her feeling, the wife redirects attention back to the husband.
- **Speech act typically responds to**:
  - **Inquiry or disengaged response** – “Aren’t you?” is more typical as a response to emotionally flat or deflective turns, serving as a probe or challenge.
- **Response focus**:
- **Shifts focus from her emotion to his emotional state** – avoids directly answering “Are you?”
- **Response strategy**:
- **Engages via emotional mirroring and deflection** – While seemingly engaging, the wife does not elaborate on her feelings but challenges him to match them.

**Step 3: Alignment Assessment**.

- **Direct alignment**:
- **No** – The response avoids the direct question posed.
- **Partial alignment**:
- **Yes** – It engages emotionally and keeps the affective tone, but sidesteps the question of her own emotion in favor of assessing his.
- **Complete misalignment**:
- **No** – It still relates to the emotional context and attempts to draw the partner into it.

**Step 4: Deflection Pattern Identification**.

- **Deflection type**:
- **Responsibility deflection** – Shifts emotional responsibility onto the husband by turning the question back.
- **Emotional redirection** – Seeks emotional validation or parity instead of providing self-validation.

**Step 5: Relationship and Context Implications**.

- **Power dynamics**:
- **Balancing or subtle reversal** – The wife redirects the emotional onus back onto the husband, asserting her desire for shared sentiment.
- **Intimacy level**:
- **Moderate to high intimacy** – The counter-question signals a desire for emotional connection, even if through indirect means.
- Conflict **indicators**:
- **Mild emotional tension or need for affirmation** – Suggests a possible gap in emotional resonance, or an effort to prompt engagement.
- **Communication style**:
- **Emotionally expressive with reciprocal demands** – The wife expresses openly and seeks similar emotional transparency from her partner.

**Conclusion Title**:

**Emotional Reciprocity Challenge: Redirecting Vulnerability to Prompt Shared Sentiment**.

SADMA OUTPUT 4.

**Analysis of Speech Act Deflection and Misalignment**.

**Step 1: Expected Response Mapping**.

- **Primary speech act**: Question seeking emotional reciprocation/confirmation (“Aren’t you [happy]?“)
- **Expected response type**: Direct affirmation or qualification of emotional state.
- **Conversational goal**: Seeking mutual emotional connection and shared experience of happiness.

**Step 2: Actual Response Analysis**.

- **Actual speech act performed**: Emphatic affirmation with intensification (“Oh, of course I am! Tremendously happy!“).
- **Speech act: the actual response typically responds to**: Direct questions about personal emotional state or wellbeing.
- **Response focus**: Directly addresses the emotional state inquiry with enthusiastic confirmation.
- **Response strategy**: Full engagement with amplification and emphasis.

**Step 3: Alignment Assessment**.

- **Direct alignment**: Yes - fully fulfills the expected speech act.
- **Partial alignment**: N/A - complete alignment achieved.
- **Complete misalignment**: No - No-response perfectly matches expectation.

**Step 4: Deflection Pattern Identification**.

- **No deflection present** - The response demonstrates perfect conversational alignment with appropriate emotional reciprocation.

**Step 5: Relationship and Context Implications**.

- **Power dynamics**: Balanced - both partners equally directing emotional sharing.
- **Intimacy level**: High comfort with emotional vulnerability and reciprocal sharing.
- Conflict **indicators: **None - harmonious emotional synchronization evident
- **Communication style**: Enthusiastic and affirming - suggests a healthy relationship dynamic.

SADMA OUTPUT 5.

**Analysis of Speech Act Deflection and Misalignment**.

**Step 1: Expected Response Mapping**.

- **Primary speech act**: Affectionate reflection/celebration of marital bond and present moment.
- **Expected response type**: Romantic reciprocation, agreement, or tender acknowledgment of their union.
- **Conversational goal**: Seeking an intimate connection and shared appreciation of their marriage.

**Step 2: Actual Response Analysis**.

- **Actual speech act performed**: Ironic complaint/lament with negative evaluation.
- **Speech act: the actual response typically responds to**: Expressions of concern, worry, or problematic situations.
- **Response focus**: Reframes marriage as a problematic outcome rather than a celebratory moment.
- **Response strategy**: Complete redirection from positive to negative framing; deflects intimacy.

**Step 3: Alignment Assessment**.

- **Direct alignment**: No - completely opposes expected romantic reciprocation.
- **Partial alignment**: No - entirely contradicts the celebratory tone.
- **Complete misalignment**: Yes - ignores intimate intent and reframes positively as negative.

**Step 4: Deflection Pattern Identification**.

- **Emotional deflection**: Avoids engaging with wife’s romantic sentiment.
- **Authenticity deflection**: Implies their marriage represents decline rather than joy.
- **Topic deflection**: Shifts from celebration of love to complaint about circumstances.

**Step 5: Relationship and Context Implications**.

- **Power dynamics**: Husband undermines wife’s emotional vulnerability with a dismissive response.
- **Intimacy level**: Low - husband retreats from an intimate moment through sarcasm.
- **Conflict indicators**: Significant tension - suggests underlying marital dissatisfaction.
- **Communication style**: Defensive and deflecting - indicates emotional unavailability or relationship strain.

SADMA OUTPUT 6.

**Analysis of Speech Act Deflection and Misalignment**.

**Step 1: Expected Response Mapping**

- **Primary speech act**: Ironic complaint/lament about their marital situation.
- **Expected response type**: Defensive justification, counterargument, or request for clarification of the negative assessment.
- **Conversational goal**: Expressing dissatisfaction or cynicism about their circumstances.

**Step 2: Actual Response Analysis**

- **Actual speech act performed**: Defensive rebuke with personal accusation about past experience.
- **Speech act the actual response typically responds to**: Mockery, dismissive humor, or perceived condescension.
- **Response focus**: Addresses perceived mockery and attributes husband’s attitude to his marital history.
- **Response strategy**: Deflects from the complaint by attacking the husband’s sincerity and past.

**Step 3: Alignment Assessment**

- **Direct alignment**: No - does not engage with his expressed dissatisfaction.
- **Partial alignment**: Yes - responds to the negative tone but misinterprets the target.
- **Complete misalignment**: No - acknowledges negativity but redirects the source.

**Step 4: Deflection Pattern Identification**

- **Responsibility deflection**: Shifts blame to husband’s past rather than addressing present complaint.
- **Emotional deflection**: Avoids engaging with his expressed dissatisfaction about their situation.
- **Temporal deflection**: References past marriage to explain present attitude.

**Step 5: Relationship and Context Implications**

- **Power dynamics**: Wife asserts vulnerability while attacking husband’s credibility.
- **Intimacy level**: Strained - reveals insecurity about being his second wife.
- **Conflict indicators**: High tension - underlying jealousy and defensive positioning evident.
- **Communication style**: Accusatory and defensive - suggests deep-seated insecurities about their relationship.
